# Idiopathic Bilateral Adrenal Haemorrhage in an Otherwise Healthy Patient

**DOI:** 10.7759/cureus.28758

**Published:** 2022-09-03

**Authors:** Clay Renwick, Alexander Yeates

**Affiliations:** 1 General Surgery, Rockhampton Hospital, Rockhampton, AUS; 2 Urology, Princess Alexandra Hospital, Brisbane, AUS

**Keywords:** abdominal pain differential diagnosis, conservative management, idiopathic adrenal haemorrhage, bilateral adrenal haemorrhage, adrenal haemorrhage

## Abstract

This is the case of a lady with a bilateral adrenal haemorrhage with no known cause. She presented with abdominal pain that progressed to back pain with rising serum lactate. The initial and repeat computerized tomography (CT) scans were unremarkable apart from small bilateral renal stones. She was ultimately treated conservatively in the hospital and discharged. On a follow-up appointment and CT scan, it was discovered that she had small bilateral adrenal haemorrhages. She was followed in the clinic until symptomatic resolution which did eventuate, and she was subsequently discharged. The case highlights that with no antecedent factors or past medical history combined with no hemodynamic instability, bilateral adrenal haemorrhages would be a diagnosis to reach but should remain in the differential.

## Introduction

An adrenal hemorrhage is rare in its own right, let alone bilateral and idiopathic. The adrenal glands are a set of endocrine glands that sit in the retroperitoneal space upon the kidneys and secrete mineralocorticoids, glucocorticoids, androgens, and catecholamines. Hemorrhage of these glands can cause symptoms of abdominal pain with respect to the bleed itself, or cardiovascular collapse stemming from a decrease/loss of hormone production/secretion and adrenal insufficiency [1]. A primary or idiopathic hemorrhage is one that doesn't have an identifiable cause. In contrast, a secondary hemorrhage can be attributed to trauma, infection, anticoagulants, pregnancy, stress, surgery, malignancy or autoimmune diseases [1]. Mortality has been found to be 15% overall and autopsy studies have found an adrenal hemorrhage in 0.3-1.8% of unselected cases and extensive bilateral hemorrhage in 15% of patients dying of shock [2]. Imaging of these hemorrhages is generally done with computed tomography with a hematoma being oval or round with possible surrounding fat stranding [3]. 

## Case presentation

This is the case of a bilateral adrenal hemorrhage found post-discharge in a patient presenting with what sounded like bilateral renal colic. A 68-year-old lady presented to a peripheral hospital with vomiting and sudden onset colicky left flank and upper quadrant pain. She denied any urinary symptoms or fever.

She has no past medical history and takes no regular medications. She was a previous smoker (abstained for 40 years) and drinks approximately 2 beers each day. Her vitals were unremarkable and her abdomen was soft but mildly tender in the left upper quadrant (LUQ) and left flank. 

With differentials at this point ranging from renal colic, pancreatitis, acute coronary syndrome, and aortic dissection, blood tests were drawn and an ECG was done. The ECG showed nonspecific T-waves and a prolonged QT interval. Given the differentials and symptoms, she was given morphine IV, metoclopramide IV, indomethacin PR, and aspirin loaded. A CT scan with contrast of the abdomen and blood tests were reassuring apart from a point of care lactate level of 3.7 and a white cell count of 15.8. Due to the lactate level and ongoing pain, she was admitted to the hospital for observation overnight. 

While in the ward, her pain escalated again, and she continued to vomit. A repeat lactate was ordered and had risen to 4.7. With a relatively benign abdominal exam and rising lactate levels, general surgery at the referral hospital was contacted and accepted a transfer with concerns for ischemic colitis. 

On arrival at the larger center the following morning, her pain began to encompass the right side of her back as well. Examination still revealed some LUQ tenderness but no flank tenderness anymore. She was subsequently started on pantoprazole for a presumed diagnosis of gastritis. 

In the evening of the first day of admission at the accepting hospital, urology became involved on the presumption that the flank pain was related to a 2mm left distal ureteric and two <3mm right lower renal calculi found on CT. She was subsequently transferred to their care for this and after 2 days of sodibic (given density of stone on CT and possible uric acid component) and indomethacin, her pain had resolved and she was discharged from the hospital.

**Figure 1:**
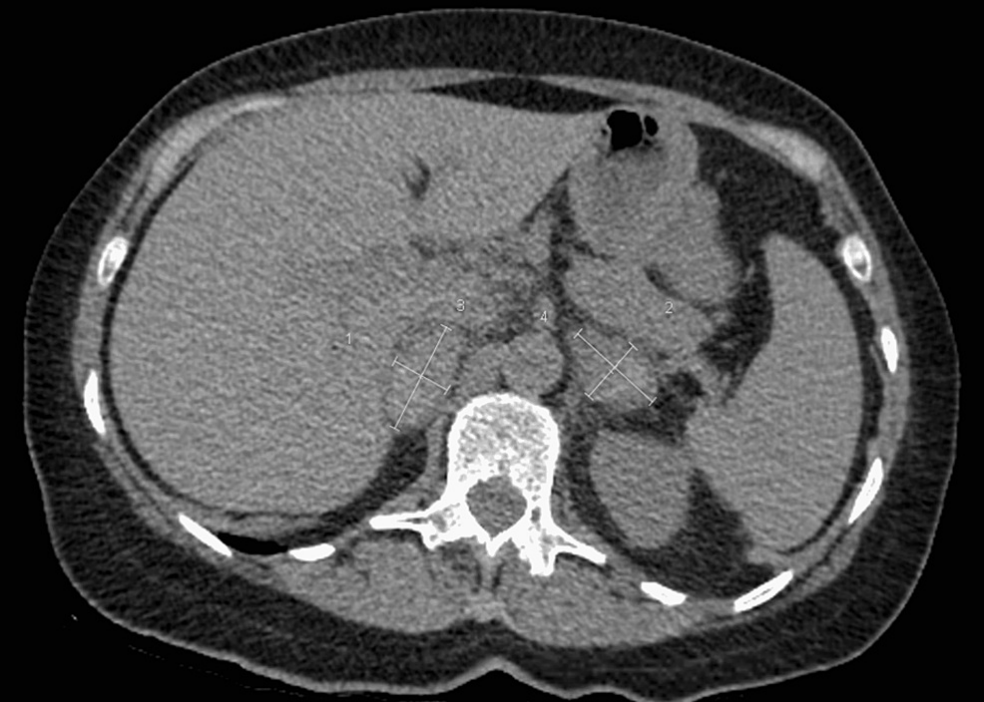
Axial view of CT scan and the bilateral adrenal hemorrhages

After 1 month of discharge from the hospital, she was seen in the urology clinic for review. Prior to arrival, she had a repeat CT abdomen without contrast (figure 1) for assessment of her ureteric stone and was found to have bilateral adrenal hemorrhages with marked enlargement of the glands (Right: 3.6 x 4.4 cm, Left: 3.2 x 4.1 cm). At this time her pain had settled and her fatigue was improving. A month later, urine catecholamines and coagulation studies were performed to assess adrenal function which was unremarkable. A further repeat CT scan (adrenal phase) was also performed at this time which showed a significant improvement in the appearance of the hematomas and was subsequently discharged from clinic follow-up. 

## Discussion

Adrenal hemorrhage has been incidentally found in as many as 1.1% of post-mortem autopsies [4]. Implicated causes of adrenal haemorrhage include trauma, antiphospholipid syndrome, sepsis/meningococcemia, and anticoagulation [5]. Other cases of bilateral adrenal haemorrhage recognize these as possible causes as well. Interestingly, anticoagulation is associated with haemorrhage for 2 opposing reasons. Firstly, due to the expected coagulopathy from anticoagulation [6]. The second is venous thrombosis secondary to heparin-induced thrombocytopenia [6]. In other patients with a preceding medical history, these conditions could potentially point the clinician to the diagnosis. However, this would not be possible if the case is truly idiopathic. 

Signs and symptoms of an adrenal hemorrhage relate to its cause, the anatomical location of adrenals, and their synthetic function. Fatigue, nausea, pain, and dizziness are recurrent themes among these patients [5]. Hypotension that is refractory to fluids and vasopressors, fever, and/or tachycardia can indicate the haemorrhage or the pre-emptive condition [5]. Deranged laboratory findings would be consistent with a loss of their hormone production and include hyponatremia with hyperkalaemia and hypoglycaemia [5]. Confirmation of an adrenal hemorrhage is largely done with imaging. Currently, CT and/or MRI are the imaging modalities commonly used [7]. In a case like this, where history, examination, and imaging do not match up with common conditions, a broadened differential and thus investigation profile is needed. We would propose that an adrenal phase CT or MRI may have been useful initially if an adrenal hemorrhage was a differential. This would, however, be on the assumption that it was a brisk enough bleed to visualize acutely. 

Literature on the management of atraumatic adrenal hemorrhage is sparse. Conservative management is the mainstay of initial management in these patients with progression to angioembolization in hemodynamically unstable patients [8,9]. This case supports findings in the case series by Ali et al. [10] who also highlight that these methods allow time for stabilization and repeat imaging to determine if there is an underlying lesion necessitating an elective laparoscopic or open adrenalectomy. 

There have been a few mentions of the phenomenon in the literature. Each of these had differing presenting complaints among themselves and compared to our patient. The first report by Dhawan et. al. [11] described a 63-year-old Caucasian male presenting with crampy umbilical pain with some epigastric tenderness. This patient’s lactic acid was elevated at 2.8 and, like our patient, had a CT in which the adrenal hemorrhages were not apparent. This 63-year-old ultimately had an MRI which highlighted the pathology and was treated with hydrocortisone. The case told of a 54-year-old woman reported by Ogino et al. [12] showed the progression from a left-sided adrenal hemorrhage to a bilateral adrenal hemorrhage on repeat imaging on the second day of being in hospital. She was treated in hospital with IV hydrocortisone for 1 week followed by a 6-month taper. Anton wrote of an 80-year-old woman who presented after a 6-week history of constitutional symptoms and was shown to have enlarged adrenals. Ultimately an MRI was used to confirm the diagnosis [13]. Initially, she was not treated with any steroid replacement, but 4 months later was noted to have adrenal insufficiency and was treated with steroids. One year following the initial event she was doing well. An otherwise well middle-aged man who presented with persistent lethargy and muscle weakness for 2 weeks was discussed by Fatima et. al. [14]. He ultimately obtained a CT scan due to an unsteady course in the hospital which showed the bleed. In this case, no indication of treatment following diagnosis was made apparent. Interestingly, this patient did not report any pain which may have prompted an earlier scan. These cases highlight the need for a high index of suspicion and either repeat imaging or a change in the imaging modality. They also make it apparent that conservative management will likely allow for recovery. 

## Conclusions

Bilateral adrenal haemorrhages are a rare cause of sudden abdominal pain and, as such, are not typically thought of in the differential, especially when there is an otherwise normal CT scan indicative of another pathology. When lactate levels are rising without a clear cause, an adrenal haemorrhage could be a possible differential. In such cases, an emergent MRI may be warranted. Similar cases of adrenal haemorrhage where there is no systemic collapse could also be managed conservatively.
